# Soil Acidification Can Be Improved under Different Long-Term Fertilization Regimes in a Sweetpotato–Wheat Rotation System

**DOI:** 10.3390/plants13131740

**Published:** 2024-06-24

**Authors:** Huan Zhang, Lei Wang, Weiguo Fu, Cong Xu, Hui Zhang, Xianju Xu, Hongbo Ma, Jidong Wang, Yongchun Zhang

**Affiliations:** 1 School of Agricultural Equipment Engineering, Jiangsu University, Zhenjiang 212013, China; czhanghuan@foxmail.com (H.Z.); fuweiguo@ujs.edu.cn (W.F.); 2 National Agricultural Experimental Station for Agricultural Environment, Luhe, Ministry of Agriculture and Rural Affairs, Institute of Agricultural Resources and Environment, Jiangsu Academy of Agricultural Sciences, Nanjing 210014, China; wanglei_njau@163.com (L.W.); cxu@jaas.ac.cn (C.X.); 1983hui@sina.com (H.Z.); xuxianju76@163.com (X.X.); mhbmhb110@126.com (H.M.); yczhang66@sina.com (Y.Z.)

**Keywords:** soil acidification, sweetpotato–wheat rotation system, soil quality improvement, long-term fertilization, organic fertilization, arid-environment eco-restoration and sustainable agriculture construction

## Abstract

Soil acidification is a significant form of agricultural soil degradation, which is accelerated by irrational fertilizer application. Sweetpotato and wheat rotation has emerged as an important rotation system and an effective strategy to optimize nutrient cycling and enhance soil fertility in hilly areas, which is also a good option to improve soil acidification and raise soil quality. Studying the effects of different fertilization regimes on soil acidification provides crucial data for managing it effectively. An eight-year field experiment explored seven fertilizer treatments: without fertilization (CK), phosphorus (P) and potassium (K) fertilization (PK), nitrogen (N) and K fertilization (NK), NP fertilization (NP), NP with K chloride fertilization (NPK1), NP with K sulfate fertilization (NPK2), and NPK combined with organic fertilization (NPKM). This study focused on the soil acidity, buffering capacity, and related indicators. After eight years of continuous fertilization in the sweetpotato–wheat rotation, all the treatments accelerated the soil acidification. Notably, N fertilization reduced the soil pH by 1.30–1.84, whereas N-deficient soil showed minimal change. Organic fertilizer addition resulted in the slowest pH reduction among the N treatments. Both N-deficient (PK) and organic fertilizer addition (NPKM) significantly increased the soil cation exchange capacity (CEC) by 8.83% and 6.55%, respectively, compared to CK. Similar trends were observed for the soil-buffering capacity (pHBC). NPK2 increased the soil K^+^ content more effectively than NPK1. NPKM reduced the sodium and magnesium content compared to CK, with the highest magnesium content among the treatments at 1.60 cmol·kg^−1^. Regression tree analysis identified the N input and soil magnesium and calcium content as the primary factors influencing the pHBC changes. Structural equation modeling showed that the soil pH is mainly influenced by the soil ammonium N content and pHBC, with coefficients of −0.28 and 0.29, respectively. Changes in the soil pH in the sweetpotato–wheat rotation were primarily associated with the pHBC and N input, where the CEC content emerged as the main factor, modulated by magnesium and calcium. Long-term organic fertilization enhances the soil pHBC and CEC, slowing the magnesium reduction and mitigating soil acidification in agricultural settings.

## 1. Introduction

Accelerated soil acidification has become an important form of soil quality degradation in farmland [1], and unreasonable fertilization is its most important cause [2]. Studying the effect and mechanism of different fertilization treatments on soil acidification can provide data support and a theoretical basis for reasonably guiding the treatment scheme for soil acidification. Currently, accelerated soil acidification is a widespread issue in Chinese farmland [3,4]. Fertilization is a significant factor contributing to the acceleration of soil acidification in agricultural fields [5,6]. This is associated with improper fertilizer application leading to the acceleration of soil nitrification [7], the leaching of basic ions [8], and the accelerated consumption of soil basic ions due to ion transfer within the crop production system [9].

Sweetpotato (*Ipomoea batatas* Lam) is an important root crop that plays an important role in ensuring food security and improving people’s nutritional status [10,11]. Compared with other crops, sweetpotato has strong drought resistance and barren tolerance [12,13] and can obtain a considerable root yield even in soil with low nutrients or fertilizer input, so sweetpotato is called an environmentally friendly low-carbon crop [14,15]. Due to the sloping terrain and heavy rainfall, hilly areas, particularly in the rainy summers in southern China, are prone to soil erosion [16]. Sweetpotato plants, with their sprawling vines and dense leaves, offer extensive ground coverage, effectively shielding the soil from the impact of rainfall and reducing surface runoff [17]. Crop rotation, particularly between sweetpotato and wheat, emerges as an effective strategy to optimize nutrient cycling and enhance soil fertility in hilly areas. Sweetpotato and wheat have distinct nutrient requirements and uptake patterns. By rotating these two crops, the residual nutrients from sweetpotato cultivation can be effectively utilized by wheat, mitigating nutrient imbalances and maximizing nutrient utilization in the soil [18,19]. However, there is limited research on the impact of different fertilization methods on the soil acidification process in the sweetpotato–wheat rotation system. Therefore, studying the effects of different fertilization practices on soil acidification in the sweetpotato–wheat rotation system is of significant practical research importance.

Chemical fertilizers, especially those containing ammonium (NH_4_), such as ammonium sulfate and urea, are widely used to enhance crop yields [20]. However, these fertilizers can lead to soil acidification through several mechanisms. The primary mechanism is nitrification, where ammonium is converted into nitrate (NO_3_) by soil bacteria, releasing hydrogen ions (H^+^) into the soil [21]. This process reduces the soil pH over time. Additionally, the use of sulfur-containing fertilizers can produce sulfuric acid upon oxidation, further contributing to soil acidification. On the other hand, organic fertilizers, including compost and manure, generally contribute to soil health by adding organic matter and improving the soil structure. The decomposition of organic matter by soil microorganisms releases organic acids and carbon dioxide (CO_2_), which can also influence the soil pH. However, the overall impact of organic fertilizers on the soil pH is often less pronounced compared to chemical fertilizers due to their buffering capacity. Organic matter increases the soil cation exchange capacity (CEC), enhancing the soil’s ability to neutralize acids and resist pH changes. Given these contrasting effects, understanding the long-term impact of these fertilization practices on the soil pH and buffering capacity is essential for developing sustainable soil management strategies. This study aims to investigate these impacts over an extended period in a controlled experimental setting [5]. Therefore, we propose the following hypotheses. (1) Long-term application of chemical fertilizers will lead to a significant decrease in the soil pH compared to organic fertilizers, primarily due to the accumulation of hydrogen ions from nitrification and the oxidation of sulfur-containing compounds. (2) Organic fertilization will result in a lesser degree of soil acidification or even an increase in the soil pH over time due to the buffering effect of the increased organic matter and enhanced microbial activity. (3) The buffering capacity of soil will be higher in plots receiving organic fertilization compared to those receiving chemical fertilization due to the enhanced cation exchange capacity and improved soil structure from the organic matter additions.

## 2. Results

### 2.1. Soil pH Changes under Different Fertilization Treatments

**Figure 1:**
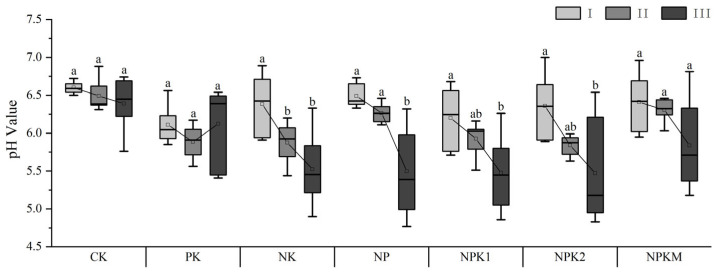
Change in the soil pH under different long-term fertilizer treatment. CK, no fertilization; PK, PK fertilization; NK, NP fertilization; NP, NP fertilization; NPK1, N and P with potassium sulfate fertilization, NPK2, N and P with potassium chloride fertilization; NPKM, N, P, and K fertilization combined with organic fertilization; values followed by different letters differ significantly among the different fertilization treatments (*p* < 0.05); I, II, and III indicate the 2012–2014, 2015–2017, and 2018–2020 years, respectively; horizontal bars within boxes represent the median. The tops and bottoms of boxes represent the 75th and 25th quartiles, respectively. The upper and lower whiskers represent the range of non-outlier data values. The square point inside the box represents the average value of the data.

Compared to the initial soil pH of 6.48, the soil pH in all the N-related fertilization treatments, except for the CK and PK treatments, exhibited a decreasing trend from 2012 to 2020 (Figure 1). The magnitude of the pH decrease, from highest to lowest, was NP (24.46%), NPK1 (24.29%), NPK2 (24.11%), NK (22.75%), and NPKM (16.97%). The corresponding decrease ranged from 0.121 to 0.225 units per year. Except for the CK and PK treatments, which showed certain fluctuations and no significant differences in the soil pH, even the NPKM treatment exhibited a rapid pH decrease at different stages. However, the soil pH decrease varied among the different N fertilization treatments during three stages: 2012–2014 (Stage I), 2015–2017 (Stage II), and 2018–2020 (Stage III). Except for the NK treatment, the pH in Stage II did not show significant differences compared to Stage I in the other treatments. Similarly, except for the NP treatment, the pH in Stage III did not exhibit significant differences compared to Stage II in the other treatments. These findings suggest that the combined application of N and K fertilizers can lead to rapid soil acidification over a short period.

### 2.2. Changes in Soil CEC under Different Fertilization Treatments

**Figure 2:**
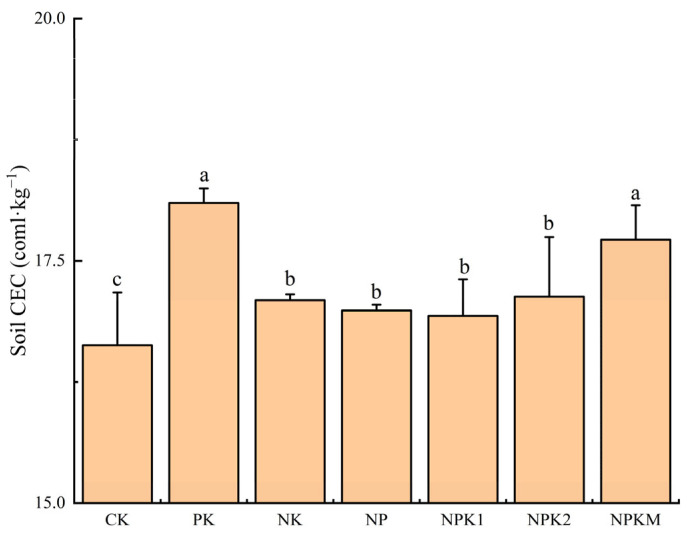
Influence of fertilizer treatment on the cation exchange capacity (CEC). CK, no fertilization; PK, PK fertilization; NK, NP fertilization; NP, NP fertilization; NPK1, N and P with potassium sulfate fertilization, NPK2, N and P with potassium chloride fertilization; NPKM, N, P, and K fertilization combined with organic fertilization; values followed by different letters differ significantly among the different fertilization treatments (*p* < 0.05); the data are expressed as means ± standard deviation.

Fertilization significantly changed the CEC of the soil (Figure 2). In comparison to the CK treatment, all the fertilization treatments resulted in a significant increase in the soil CEC. The soil CEC content was highest in the PK treatment, reaching 18.2 cmol·kg^−1^, followed by the NPKM treatment at 17.7 cmol·kg^−1^. Both of these treatments showed a significant increase compared to the other fertilization treatments. This indicates that without N fertilizer treatments or those combining NPK fertilizers with additional organic fertilizers, the soil CEC content significantly increases.

### 2.3. Changes in Exchangeable Base Cation Ion Content in Soil under Different Fertilization Treatments

Fertilization significantly changed the content of exchangeable base cations in the soil (Table 1). The results show that the PK treatment, which received no N fertilization over the long term, exhibited the highest levels of Na^+^, K^+^, Ca^2+^, and Mg^2+^, with the highest total exchangeable base cation content (ECEC). For K^+^, the highest levels were observed in the PK and NK treatments, which received long-term K fertilization, while the NK treatment without P fertilizer input showed the lowest levels of Ca^2+^ and Mg^2+^ ions. Significant differences were observed in the K^+^, Ca^2+^, and Mg^2+^ content between the NPK1 treatment, which received long-term potassium chloride fertilization, and the NPK2 treatment, which received long-term potassium sulfate fertilization. Specifically, the NPK1 treatment had significantly higher Ca^2+^ and Mg^2+^ content than the NPK2 treatment, while the K^+^ content showed the opposite trend. The NPKM treatment, with organic fertilizer input, exhibited higher levels of K^+^, Ca^2+^, and Mg^2+^, with the lowest Na^+^ content. The total content of exchangeable base cations (ECEC) in the different treatments showed that the NK treatment without P fertilization had the lowest content, significantly lower than the other treatments. The NPK2 treatment, which received potassium chloride fertilization, ranked second in terms of the ECEC, while the other treatments showed no significant differences.

### 2.4. Changes in Soil-Buffering Capacity under Different Fertilization Treatments

**Figure 3:**
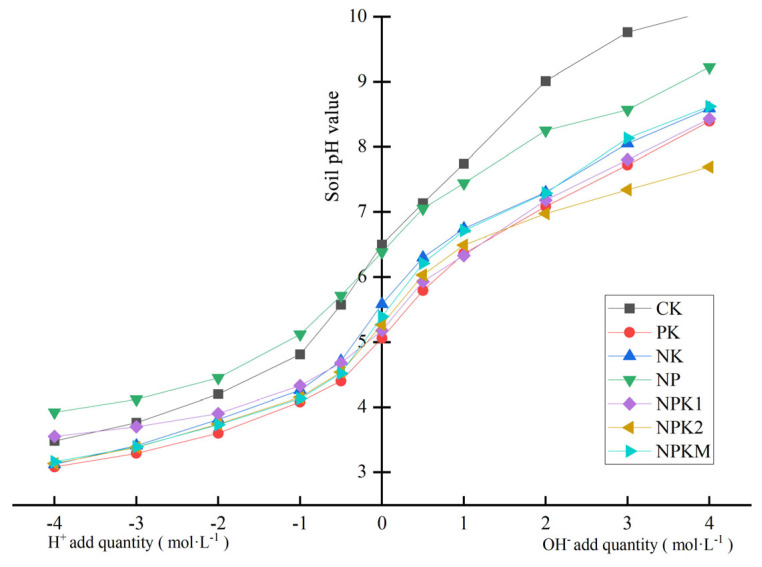
Soil titration curves under different fertilization treatments. CK, no fertilization; PK, PK fertilization; NK, NP fertilization; NP, NP fertilization; NPK1, N and P with potassium sulfate fertilization, NPK2, N and P with potassium chloride fertilization; NPKM, N, P, and K fertilization combined with organic fertilization.

The soil acid–base titration curves under different fertilization treatments are shown (Figure 3). The results indicate that the soil pH exhibited linear changes in the pH range of 4.0 < pH < 7.5. Linear fitting of the mutation region revealed the soil-buffering capacity under different fertilization treatments (Table 2). The results suggest that long-term fertilization increased the soil-buffering capacity. The CK treatment had the lowest soil-buffering capacity (18.87 mmol·kg^−1^), significantly lower than the PK treatment without N fertilization and the NPKM treatment with additional organic fertilizer. However, there were no significant differences between the CK treatment and the other fertilization treatments. Among the different fertilization treatments, NPKM had the highest soil-buffering capacity, with a pHBC value of 24.57 mmol·kg^−1^, followed by the PK treatment, with a pHBC value of 24.42 mmol·kg^−1^. Nevertheless, the variations among the fertilization treatments did not reach statistical significance.

### 2.5. Correlation Analysis of Soil Physicochemical Properties

Pearson correlation analysis reveals highly significant positive correlations between the soil pH and pHBC, as well as between the soil pH and CEC. On the other hand, the soil pH shows significant negative correlations with the soil AN, and K^+^ content (Table 3). This suggests that an increase in the soil cation exchange capacity and buffering capacity significantly slows down soil acidification. Simultaneously, the soil pH is strongly negatively correlated with the soil available N and organic matter content, indicating that the decrease in the soil pH is associated with the nitrogen input and the corresponding increase in the amount of returned straw. The soil organic matter content is positively correlated with the pHBC, K^+^, and pHBC, while it shows no significant correlation with the Ca^2+^, Na^+^, and available P content. The pHBC is significantly positively correlated with the CEC, soil organic matter, and soil Ca^2+^ and Mg^2+^ content.

**Figure 4:**
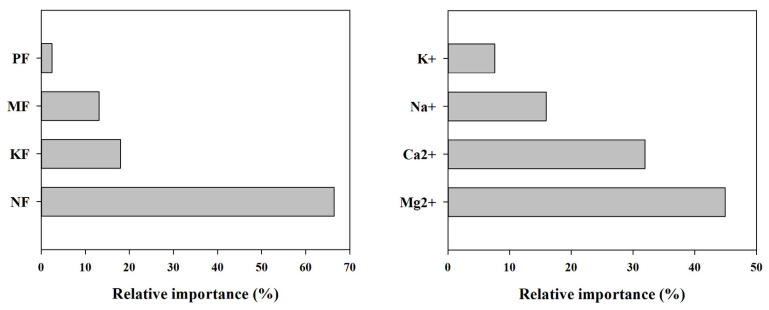
Relative importance of factors affecting the soil pHBC change. NF, nitrogen fertilization; KF, potassium fertilization; MF, organic fertilization; PF, phosphorus fertilization.

Using the random forest method to evaluate the relative importance of different fertilization treatments on the soil-buffering capacity (Figure 4), it is evident that the fertilizers have varying degrees of impact on the change in the soil-buffering capacity, with the impact ranking as follows: N fertilization (NF) > K fertilization (KF) > organic fertilization (MF) > P fertilization (PF). Among these, the application of N fertilization has the highest relative importance in influencing the soil-buffering capacity, accounting for 66.45%. In comparison, KF, MF, and PF have lower relative importance, with values of 18.01%, 13.10%, and 2.44%, respectively. When comparing the relative importance of different ions in causing changes in the buffering capacity, magnesium ion (Mg^2+^) has the highest relative importance among the cations, accounting for 44.96%, followed by calcium ions (Ca^2+^) and sodium ions (Na^+^), with relative importance values of 31.95% and 15.95%, respectively. The potassium ion (K^+^) has the lowest relative importance at 7.85%, indicating that Mg^2+^ and Ca^2+^ ions are the main cations influencing changes in the soil-buffering capacity in the experiment.

**Figure 5:**
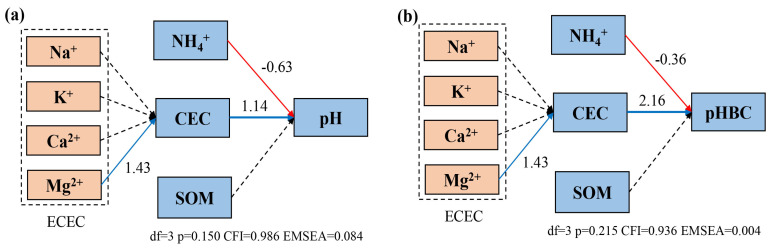
Structural equation modeling of the pH, pHBC, CEC and each cation. (**a**): indirect effect of cationic ions on soil pH; (**b**) indirect effect of cationic ions on soil pHBC. The red and blue arrows indicate the positive and negative relationships between the indicators. The number above the arrow indicates the path coefficient. The solid line and the dashed line represent the significant path and the insignificant path, respectively (*p* < 0.05). The model fits the data well, that is, df = 8, *p* = 0.164, CFI = 0.967, RMSEA = 0.024.

The soil pH is primarily influenced by processes such as leaching and the formation of complex salts. Exchangeable cations in the soil exist mainly in the forms of Na^+^, Mg^2+^, Ca^2+^, and K^+^, and the concentrations of these cations are key factors affecting changes in the soil cation exchange capacity (CEC). To clarify the impact of various factors on soil acidification, a structural equation model was established to analyze relevant indicators. Based on indicators such as the degrees of freedom (df), P-values, RMSEA, CFI, etc., the model demonstrated a good fit. Through this model (Figure 5), it is evident that Mg^2+^ in yellow–brown soil with a pH range of 5.0 to 6.2 has a direct positive impact on the CEC, with a path coefficient of 1.43. The soil CEC, in turn, influences the pH changes through a positive correlation with the pHBC. The soil organic matter content has a positive impact on both the soil pHBC and the pH, with path coefficients of 0.51 and 0.05, respectively. Simultaneously, the NH_4_^+^ in the soil has direct negative effects on both the pH and the pHBC, with path coefficients of −0.28 and −0.67, respectively.

## 3. Discussion

### 3.1. Relationship between Crop Rotation Systems and Soil Acidification Characteristics

There are differences in the soil acidification characteristics and rates under different cropping systems [22]. In this study, the soil of a sweetpotato–wheat rotation system under long-term different fertilization treatments showed accelerated acidification. Especially in the treatment of applying nitrogen fertilizer, the acidification rate reaches 0.121~0.225 units per year, which is much higher than that of rice and vegetable rotation and long-term rice planting; the latter are 0.076 units per year and 0.007 units per year, respectively, which may be related to the accumulation of ammonia nitrogen, the reduction of carbon dioxide to methane, and the oxidation and reduction of iron and sulfur caused by flooding farming, which makes the soil pH gradually stable with the farming time. This may be related to the accumulation of ammonia nitrogen, the reduction of carbon dioxide to methane, and the oxidation and reduction of iron and sulfur caused by the flooding environment, which makes the soil pH gradually stable between 6 and 7 with the tillage time [23]. Compared with simple flooded farming, it has a longer drainage period. Because the soil in the dry season is under aerobic conditions, nitrification and leaching of products are easier. Therefore, adding nitrogen fertilizer to paddy-upland farming and simple dry farming will accelerate soil acidification [24]. Guo et al. (2010) found that the soil acidification rate of corn–wheat rotation was significantly higher than that of rice–wheat rotation and rice–rice rotation, and its acid mainly came from the application of nitrogen fertilizer [3]. Different plant species can significantly impact the soil pH and contribute to either the mitigation or exacerbation of soil acidification through various mechanisms. Plants release root exudates, which include organic acids, amino acids, sugars, and other compounds. These exudates can alter the soil pH in the rhizosphere. For instance, the exudation of organic acids like citric acid and malic acid can lower the soil pH by increasing the concentration of hydrogen H^+^ in the soil [25]. Conversely, some plants release alkaline root exudates that can raise the soil pH. Plants take up various nutrients from the soil, and the form in which they take up these nutrients can affect the soil pH. For example, the uptake of NH_4_^+^ by plants typically leads to soil acidification because plants release H^+^ ions to balance the charge [26]. On the other hand, NO₃⁻ uptake is usually associated with the release of hydroxyl ions or bicarbonate ions, which can increase the soil pH. The quality of plant litter, including its chemical composition, affects the soil pH during decomposition. Litter high in base cations (Ca^2+^, Mg^2+^, K^+^, and Na^+^) tends to reduce the soil acidity upon decomposition because these cations can neutralize the acidic components in the soil. In contrast, litter high in acid-forming elements, such as sulfur and nitrogen, can contribute to soil acidification [27]. Although the amount of acid produced by the transfer of base ions caused by crop planting increased slightly, the difference was not significant, which indicated that the acceleration of soil acidification under sweetpotato–wheat rotation might be related to the acid caused by the application of nitrogen fertilizer in dry land. Person analysis also showed that the soil pH was negatively correlated with the soil available N and mineral N content, which was consistent with the related results of a previous study [28].

### 3.2. Relationship between Different Fertilization Treatments and Soil Acidification Characteristics

N fertilizer application, crop uptake of cations, and precipitation are considered the three major driving factors of soil acidification [29]. Continuous nitrification is the main cause of soil acidification when a large amount of N fertilizer is applied. However, the application of phosphate fertilizer and potassium fertilizer will affect the content of basic ions in the soil [30]. The cation exchange capacity (CEC) of soil can be used as an important index to express the buffering capacity of soil. The increase in the CEC can effectively improve the acid-buffering capacity (pHBC) of soil and then play a regulatory role in the acidification process of soil [31,32]. In this study, the soil Ca^2+^ in the NK treatment without phosphorus fertilizer was significantly lower than in the other treatments with phosphorus fertilizer. This is related to the high Ca^2+^ content in superphosphate, and the application of P fertilizer can compensate for the portion of Ca^2+^ taken away by crops through selective absorption, thus enhancing the soil’s acid-buffering capacity. Similar studies also show that the application of calcium superphosphate can improve the soil pH and acid-buffering capacity [33,34]. The structural equation model indicates a significant impact of the pHBC on the soil pH. The influence of P fertilizer addition on soil acidification may be related to the impact on the soil acid-buffering system, with no direct effect on the soil pH. The pH changes in the NPK1 and NPK2 treatments with potassium chloride and potassium sulfate are similar, with a greater decrease in the pH in the NPK2 treatment with potassium sulfate fertilization. This may be attributed to the long-term input of chloride ions, leading to a significant reduction in the exchangeable cations (ECEC) in the soil compared to the potassium sulfate input [35]. In comparison to sulfate ions, which are prone to specific adsorption, research by Tan et al. and others indicates that over 85% of chloride ions applied to the soil are lost through water runoff [36]. The addition of organic fertilizer on the basis of chemical fertilizers reduced the soil acidification rate. Compared to the NPK1 and NPK2 treatments with potassium chloride and potassium sulfate, the pH increased by 0.18 and 0.19 units, respectively. However, compared to the CK treatment, soil acidification still showed an accelerating trend. This suggests that adding organic fertilizer on the basis of chemical fertilizers has a certain mitigating effect on soil acidification, but the effect is limited. This is due to the limited alkaline substances in organic fertilizers, and the N in organic fertilizers still participates in the acid-producing processes in the soil. Studies indicate that although the addition of organic fertilizer has a certain positive effect on the soil pH in strongly acidic red soils, long-term experiments in paddy soils in the Taihu Lake region also show that the addition of pig manure accelerates soil acidification. This is related to the properties of organic fertilizers and the initial pH of the soil. Organic fertilization presents a viable strategy for mitigating soil acidification through several mechanisms. The decomposition of organic matter releases humic substances, which have a high CEC. This enhances the soil’s ability to buffer pH changes by neutralizing both acidic and alkaline inputs [37]. The presence of humic substances helps stabilize the soil pH, reducing the likelihood of drastic pH fluctuations. The decomposition of organic materials releases essential nutrients, including basic cations such as Ca^2+^, Mg^2+^, K^+^, and Na^+^. These cations can displace H^+^ and Al^3+^ from soil colloids, which are primary contributors to soil acidity. By displacing H^+^ and Al^3+^ ions, these basic cations help increase the soil pH and reduce the soil acidity [38]. Organic fertilizers provide a balanced nutrient supply, reducing the dependency on acidifying chemical fertilizers. The nitrogen in organic fertilizers is released slowly through mineralization, which reduces the risk of soil acidification associated with the rapid release of nitrogen from chemical sources. Additionally, the presence of other nutrients in organic fertilizers can reduce the need for additional inputs, further decreasing the potential for acidification [39]. These mechanisms provide a comprehensive approach to maintaining soil health and ensuring sustainable agricultural productivity. As the challenges of soil acidification become more pressing due to intensive farming practices, integrating organic fertilization methods becomes increasingly vital.

## 4. Materials and Methods

### 4.1. Experiment Site Description

The experiment was conducted at the Luhe Experimental Base of the Jiangsu Academy of Agricultural Sciences (118°37′ E, 32°28′ N), situated in Nanjing, Jiangsu Province, China. The site represents a subtropical monsoon climate, with an average annual temperature of 15.6 °C and an average annual precipitation of 1100 mm. The soil type is classified as yellow–brown soil. At the beginning of the experiment, the topsoil’s basic physicochemical properties were as follows: pH value of 6.48, available N of 106 mg·kg^−1^, available P of 8.4 mg·kg^−1^, available K of 115 mg·kg^−1^, and organic matter content of 13.3 g·kg^−1^.

### 4.2. Experimental Design

This experiment was a long-term field trial conducted over multiple years, initiated in 2012, using a wheat–sweetpotato rotation system. The experiment consisted of 7 treatments, namely: (1) CK (without fertilization); (2) PK (PK fertilization); (3) NK (NK fertilization); (4) NP (NP fertilization); (5) NPK1 (N and P with potassium sulfate fertilization); (6) NPK2 (N and P with potassium chloride fertilization); and (7) NPKM (N, P, and K fertilization combined with organic fertilization). Each treatment included three replicates and random block designs; each plot area was 33.3 m^2^ (5 m × 6.66 m). The planting system involved a rotation of winter wheat (*Triticum aestivum*)–summer sweetpotato (*Ipomoea batatas* Lam). The wheat variety ‘Ningmai 26’ was sown in November 2019 with a planting density of 112.5 kg·ha^−1^ and harvested in May 2020. The sweetpotato variety ‘Ningzi 4’ was planted in June 2020 and harvested in October, 2020, with a planting density of 45,000 ha^−1^. The fertilizer application rates for each season and treatment are detailed in Table 4. Briefly, the chemical N, P, and K fertilizers were urea (46% N), single superphosphate (5% P), and potassium sulfate (41% K), respectively. The organic fertilizer used in this study was swine manure obtained from nearby dairy industries and composted for at least four months. The organic fertilizer nutrient concentrations were 1.95–2.11% N, 0.55–0.66% P, and 0.96–1.26% K. The amount of organic fertilizer used in the wheat season was 3150 kg ha^−1^, and that in sweetpotato season was 1800 kg ha^−1^. In the wheat season, the N, P, and K in the organic fertilizer accounted for 30%, 50%, and 50% of the total input of N, P, and K, respectively. In the sweetpotato season, the N, P, and K in the organic fertilizer accounted for 30%, 45%, and 15% of the total input of N, P, and K, respectively.

### 4.3. Soil Sample Collection and Physicochemical Analysis

The soil sample collection procedure followed the method used in our previous research [40]. Specifically, soil samples were collected from each plot in June and October 2020 (after the sweetpotato were harvested). In each plot, plum blossom sampling technology was adopted to ensure the representativeness of the soil samples [41]. We collected a total of 21 samples, including 7 fertilization treatments and 3 replicates. After sieving through a 2 mm sieve, the samples were divided into two sub-samples. Each soil sample was air-dried to determine the physicochemical properties of the soil. The pH value of the soil was measured using a composite electrode pH meter with a soil-to-water ratio of 2.5:1 [42]. The soil organic carbon (SOC), total N, and alkali-hydrolyzable N were determined by K_2_Cr_2_O_7_ oxidation–reduction titration, the Kjeldahl method, and the alkaline hydrolysis diffusion method, respectively [43]. The available P concentrations in the soil were determined through the molybdenum blue colorimetric method after extraction with a 0.5 M NaHCO_3_ solution at a pH of 8.5 [44]. For the determination of the ammonium nitrogen (NH_4_-N) and nitrate nitrogen (NO_3_-N) concentrations in the soil, a 0.01 M calcium chloride solution was used for extraction, and the analysis was conducted using a SMART CHEMTM 200 [45]. The soil-buffering capacity (pHBC) was determined using an acid–base titration method with water extracted without CO_2_ distillation [46]. The cation exchange capacity (CEC) was determined using the ammonium acetate exchange method [47]. The exchangeable cations (Ca^2+^, Mg^2+^, K^+^, Na^+^) were extracted using 1 M ammonium acetate (NH_4_OAc) at pH 7.0 [48,49]. The concentrations of Ca^2+^ and Mg^2+^ in the extract were determined using atomic absorption spectroscopy. K^+^ and Na^+^ concentrations were measured using flame photometry.

### 4.4. Statistical Analysis

A one-way ANOVA, followed by Duncan’s test, were performed to assess the significance of the mean differences among the various treatments at *p* < 0.05. Pearson correlation analysis was performed to investigate the relationships between the different soil variables. The data were organized and tabulated using Excel 2019. Graphs were generated using Origin Pro 2021. A relative importance analysis was performed using the random forest method, and the significance of each predictor was assessed with the “randomForest” and “rfPermute” R software packages (R.4.3.1) [50,51]. Differential tests and correlation analyses were performed using IBM SPSS 25 (SPSS Inc., New York, NY, USA). Structural equation modeling (SEM) is a multivariate statistical model that is a synthesis of factor analysis and path analysis. The best-fit model was determined using generalized least squares, and the overall suitability of the model fit was assessed based on the chi-square test (*p* > 0.05), comparative fit index (>0.90), and root mean square errors of approximation (<0.08).

## 5. Conclusions

We have investigated soil acidification and its characteristics under different fertilization treatments over an eight-year period in the sweetpotato–wheat rotation system. The results indicate that the soil acidification under the sweetpotato–wheat rotation is accelerating, and its acidification rate is much higher than that under the rice–wheat rotation. The application of chemical N fertilizer is the primary cause of soil acidification. The soil pH is mainly regulated by the soil pH-buffering capacity (pHBC) and ammonium N content, while changes in the cation exchange capacity (CEC) induced by Mg^2+^ and Ca^2+^ are the dominant factors affecting the pHBC. Long-term application of organic fertilizer can effectively increase the soil pHBC but has a limited impact on raising the soil pH. Reducing the fertilizer input, especially chemical N fertilizer, and appropriately increasing the organic fertilizer application can alleviate soil acidification.

## Figures and Tables

**Table 1:** Contents of each cation under different fertilization treatment.

Treatment	Exchangeable Sodium (Na^+^)	Exchangeable Potassium (K^+^)	Exchangeable Calcium (Ca^2+^)	Exchangeable Magnesium (Mg^2+^)	ECEC
CK	0.125 a	0.21 e	4.23 a	2.14 a	13.1 a
PK	0.128 a	0.61 a	4.65 a	1.31 d	12.7 a
NK	0.116 bc	0.64 a	1.94 c	1.06 f	6.7 c
NP	0.117 bc	0.17 e	4.43 a	1.51 c	12.2 a
NPK1	0.123 ab	0.31 d	4.68 a	1.52 c	12.8 a
NPK2	0.124 a	0.46 b	2.87 b	1.19 e	8.7 b
NPKM	0.111 c	0.40 c	4.25 a	1.66 b	12.3 a

CK, no fertilization; PK, PK fertilization; NK, NP fertilization; NP, NP fertilization; NPK1, N and P with potassium sulfate fertilization, NPK2, N and P with potassium chloride fertilization; NPKM, N, P, and K fertilization combined with organic fertilization; ECEC, effective cation exchange capacity; Different lowercase letter indicated significant difference (*p* < 0.05) among fertilization treatment.

**Table 2:** Statistical analysis of the soil pHBC under different fertilization treatment.

Treatment	Slope	Intercept	R^2^	pHBC (mmol·kg^−1^·pH^−1^)
CK	1.118	6.570	0.9725	18.87 b
PK	1.310	5.518	0.9876	24.42 a
NK	1.370	5.392	0.9859	21.64 ab
NP	1.196	6.340	0.9931	21.28 ab
NPK1	1.190	5.138	0.9866	21.52 ab
NPK2	1.050	5.290	0.9854	22.86 ab
NPKM	1.234	5.296	0.9888	24.57 a

CK, no fertilization; PK, PK fertilization; NK, NP fertilization; NP, NP fertilization; NPK1, N and P with potassium sulfate fertilization, NPK2, N and P with potassium chloride fertilization; NPKM, N, P, and K fertilization combined with organic fertilization; ECEC, effective cation exchange capacity; Different lowercase letter indicated significant difference (*p* < 0.05) among fertilization treatment.

**Table 3:** Pearson correlation analysis between each factor.

	pH	pHBC	CEC	ECEC	Na^+^	K^+^	Ca^2+^	Mg^2+^	AN	NH_4_^+^	NO_3_^−^	SOM
pH	1	0.964 **	0.840 **	NS	NS	0.633 *	NS	NS	−0.858 **	NS	NS	−0.785
pHBC		1	0.810 **	NS	NS	NS	0.643 **	NS	NS	NS	NS	0.800 *
CEC			1	NS	NS	NS	0.518 *	NS	NS	NS	NS	0.503 *
ECEC				1	NS	0.658 **	0.980 **	0.726 **	−0.449 *	−0.548 *	NS	NS
Na^+^					1	NS	NS	NS	−0.541 **	NS	−0.620 *	NS
K^+^						1	−0.607 **	−0.449 *	−0.811 **	NS	0.439 *	0.487 *
Ca^2+^							1	0.579 **	NS	−0.533	NS	NS
Mg^2+^								1	0.442 *	NS	NS	−0.616 *
AN									1	NS	0.515 *	0.859 **
NH_4_^+^										1	NS	NS
NO_3_^−^											1	NS
SOM												1

* and ** indicate that the correlation is significant at the 0.05 and 0.01 levels, respectively; NS, no significant correlation.

**Table 4:** Arrangement table of the fertilizer application for the different fertilizer treatment experiment.

Treatment	Wheat (kg·ha^−1^)				Sweetpotato (kg·ha^−1^)			
	N	P	K	Organic Fertilizer	N	P	K	Organic Fertilizer
CK	0	0	0	0	0	0	0	0
NP	210	39	0	0	120	26	0	0
NK	210	0	75	0	120	0	149	0
PK	0	39	75	0	0	26	149	0
NPK1	210	39	75	0	120	26	149	0
NPK2	210	39	75	0	120	26	149	0
NPKM	210	39	75	3060	120	26	149	2040

CK, no fertilization; PK, PK fertilization; NK, NP fertilization; NP, NP fertilization; NPK1, N and P with potassium sulfate fertilization, NPK2, N and P with potassium chloride fertilization; NPKM, N, P, and K fertilization combined with organic fertilization.

## Data Availability

Data are contained within the article.
